# Upregulation of Somatostatin Receptor Expression in Lactating Breast Shown in ^99m^Tc-HYNIC-TOC Scintigraphy

**DOI:** 10.3390/diagnostics16142251

**Published:** 2026-07-18

**Authors:** Mengyao Jia, Jie Feng, Yaping Luo

**Affiliations:** Department of Nuclear Medicine, State Key Laboratory of Common Mechanism Research for Major Diseases, Chinese Academy of Medical Sciences and Peking Union Medical College Hospital, Beijing 100730, China

**Keywords:** somatostatin receptor, 99mTc-HYNIC-TOC, lactation, breast scan abnormal

## Abstract

A 31-year-old female patient with a 4-year history of multiple endocrine neoplasia type 1 (MEN1) presented with abdominal pain and weight loss that she had been experiencing over the past 6 months. Somatostatin receptor (SSTR) scintigraphy using 99mTc-HYNIC-TOC revealed increased uptake in the known pancreatic neuroendocrine tumor as well as diffuse increased uptake in bilateral lactating breasts. This case highlights physiological upregulation of somatostatin receptor expression in a lactating breast.

**Figure 1:**
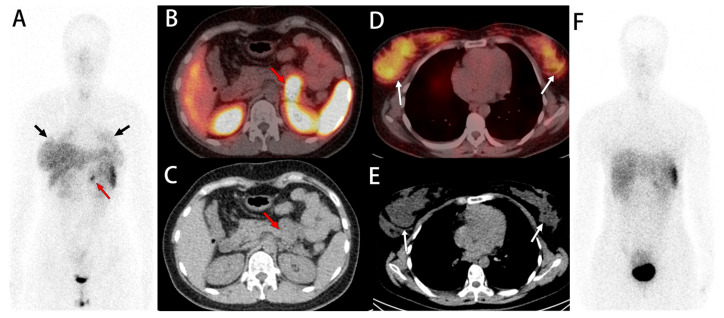
Case reports have demonstrated increased radiotracer uptake in lactating breasts for various agents, including 99mTc-pertechnetate [1], radioiodine [2], 18F-FDG [3], 99mTc-MIBI [4], 99mTc-TF, 123I-BMIPP [5], 18F-FCH [6], 11C-Methionine [7], and 99mTc /68Ga-FAPI [8,9]. The increased radioactivity in the lactating breast is possibly due to upregulation of the sodium-iodide symporter, hypermetabolic status and energy expenditure during milk production, or hyperplasia of breast and fibro-glandular tissue; the latter two mechanisms may also partially explain our case, whereas the most important and direct mechanism is likely hormone-receptor-mediated uptake. Breast uptake of 68Ga-DOTA-TATE (SSTR2-selective) in nursing women has been previously reported [10,11], suggesting physiological upregulation of SSTR expression in lactating breasts. The present case, in which 99mTc-HYNIC-TOC (targeting SSTR2/5) was used, provides further evidence supporting this phenomenon. This not only addresses the gap in identifying physiological artifacts with a SPECT tracer but also verifies the universality of SSTR expression in lactating breast tissue. Prior work established low and heterogeneous SSTR2 expression in normal breast tissue, a finding that greatly underpins these imaging observations [12]. Of note, the SSTR2 subtype, as the principal mediator of somatostatin effects, is overexpressed at a higher level in breast cancer [13,14]; therefore, increased breast uptake in SSTR imaging should increase the possibility of breast cancer, along with inflammatory conditions like mastitis, benign proliferative, or other entities featuring mechanisms overlapping with those of lactation. The present case illustrates that a lactating breast may also exhibit increased radioactivity in SSTR imaging, and thus physiological uptake during lactation should be included in the differential diagnosis when diffuse breast uptake is encountered in SSTR imaging, especially in lactating female patients. Panels (**A**–**E**) show images with 99mTc-HYNIC-TOC obtained recently, while Panel (**F**) shows an image acquired 3 years ago with a comparable imaging protocol, injected activity, and acquisition parameters. A 31-year-old female patient with a 4-year history of multiple endocrine neoplasia type 1 (MEN1) presented with parathyroid adenoma and multifocal neuroendocrine tumors (NETs) involving the gall bladder, gastric body, and pancreas. All lesions were surgically resected except for the asymptomatic pancreatic tumor. She complained of abdominal pain and weight loss over the past 6 months and was referred for somatostatin receptor (SSTR) scintigraphy using 99mTc-HYNIC-TOC to monitor the NET lesions. As shown in the anterior planar image obtained 4 h post-injection (**A**), a nodular focal lesion with increased uptake was noted in the left upper quadrant of the abdomen (**A**, red arrow), which was further confirmed via SPECT/CT to be a pancreatic tumor located in the body of the pancreas (**B**,**C**, red arrow). Additionally, diffuse increased uptake in the bilateral thoracic region, protruding beyond the thoracic contour with right-sided predominance, can be observed in the planar image (**A**, black arrows). SPECT/CT images of the chest confirmed that this radioactivity was attributable to the asymmetrical diffuse uptake in bilateral breasts (**D**,**E**, white arrows). Further history-taking revealed that the patient had been lactating for the past 7 months, suggesting overexpression of SSTR in lactating breasts. The 99mTc-HYNIC-TOC scintigraphy that was performed 3 years earlier (**F**) showed no abnormal uptake in the breast.

## Data Availability

The original contributions presented in this study are included in the article. Further inquiries can be directed to the corresponding author.
